# Impact of Androgens on Inflammation-Related Lipid Mediator Biosynthesis in Innate Immune Cells

**DOI:** 10.3389/fimmu.2020.01356

**Published:** 2020-06-30

**Authors:** Simona Pace, Oliver Werz

**Affiliations:** Department of Pharmaceutical and Medicinal Chemistry, Institute of Pharmacy, Friedrich Schiller University, Jena, Germany

**Keywords:** neutrophils, lipid mediators, 5-lipoxygenase, testosterone, leukotriene, sex

## Abstract

Rheumatoid arthritis, asthma, allergic rhinitis and many other disorders related to an aberrant immune response have a higher incidence and severity in women than in men. Emerging evidences from scientific studies indicate that the activity of the immune system is superior in females and that androgens may act as “immunosuppressive” molecules with inhibitory effects on inflammatory reactions. Among the multiple factors that contribute to the inflammatory response, lipid mediators (LM), produced from polyunsaturated fatty acids, represent a class of bioactive small molecules with pivotal roles in the onset, maintenance and resolution of inflammation. LM encompass pro-inflammatory eicosanoids and specialized pro-resolving mediators (SPM) that coexist in a tightly regulated balance necessary for the return to homeostasis. Innate immune cells including neutrophils, monocytes and macrophages possess high capacities to generate distinct LM. In the last decades it became more and more evident that sex represents an important variable in the regulation of inflammation where sex hormones play crucial roles. Recent findings showed that the biosynthesis of inflammation-related LM is sex-biased and that androgens impact LM formation with consequences not only for pathophysiology but also for pharmacotherapy. Here, we review the modulation of the inflammatory response by sex and androgens with a specific focus on LM pathways. In particular, we highlight the impact of androgens on the biosynthetic pathway of inflammation-related eicosanoids in innate immune cells.

## Introduction

Inflammation is a protective response mounted by the host immune system against invading pathogens, foreign bodies, or injuries. It is a highly coordinated, active process in order to remove the harmful stimulus and to repair damaged tissues for reestablishing homeostasis. But if an acute inflammatory response fails to resolve, it may contribute to organ pathologies and promote many widespread chronic inflammatory clinical phenotypes such as arthritis, neurodegenerative diseases, asthma, allergy, diabetes, organ fibrosis, cancers, and various cardiovascular disorders (1). Among the plethora of mediators that regulate inflammation in a spatial and temporal fashion, lipid mediators (LM) are crucial molecules that can act at the four major stages of the inflammatory process: initiation, maintenance, resolution, and the return to homeostasis (2, 3). LM encompass a huge class of highly bioactive oxygenated polyunsaturated fatty acids (PUFAs) that play broad and major roles in health and diseases. Here, we refer to LM that are biosynthesized on demand mainly from the ω-6 PUFA arachidonic acid (AA), and the ω-3 PUFAs eicosapentaenoic acid (EPA) and docosahexaenoic acid (DHA) via initial conversion by cyclooxygenases (COX), lipoxygenases (LOX) and monooxygenases (i.e., CYP450 enzymes) (4, 5). These enzymes may partially act in conjunction, and can be coupled to epoxide hydrolases, glutathione transferase and other enzymes to produce defined LM with specific biological functions by acting at select G protein-coupled receptors (GPCR) or nuclear receptors (6, 7). Whereas, most of the AA-derived prostaglandins (PG) and leukotrienes (LT) formed by the COX and 5-lipoxygenase (5-LOX) pathway, respectively, exhibit rather pro-inflammatory properties (3, 8), the so-called specialized pro-resolving mediators (SPM) including lipoxins (LX), protectins (PD), resolvins (Rv), and maresins (MaR) are generated from AA, EPA and DHA with CYPs and 12/15-LOX as key enzymes and promote resolution of inflammation (2, 9).

The responses of the innate and adaptive immune system to foreign and self-antigens differ between females and males, where both genes (on X- and Y-chromosomes) and sex hormones are involved (10, 11). Thus, the age is an important variable where certain immune responses dominate in boys vs. girls before puberty with an opposite bias in adults where the immune system is more pronounced in females (11, 12). Most sex-based immunological differences are obvious after puberty and before reproductive senescence, indicating a crucial impact of sex hormones. These sex differences contribute to divergences in the incidence of autoimmune diseases and other inflammation-related malignancies with implications for both disease pattern and therapy (11). The sex steroids are known to have direct effects on the immune response. They comprise estrogens, progesterone and androgens and are mainly biosynthesized by the gonads of adult females and males. Estrogens (i.e., estrone, estriol, and the most bioactive estradiol) are produced by ovarian granulosa cells in females by the action of the aromatase complex from androstenedione or testosterone. Effects of estrogens on the immune system have often been connected to a pro-inflammatory phenotype (13) although their “stimulatory” action on macrophages results into promotion of the resolution of inflammation (14). Progesterone, besides its well-known essential activity in pregnancy, has been reported to have anti-inflammatory properties by influencing the immune system (15). More recent findings showed that progesterone suppresses LT biosynthesis in human monocytes by inhibition of 5-LOX activity (16). Androgens exert several inhibitory effects on immune cell activity with predominantly anti-inflammatory properties (17). Thus, the androgens testosterone and 5α-dihydrotestosterone (5α-DHT) are highly potent sex hormones that affect a variety of innate and adaptive immune responses with suppressive effects on macrophages, neutrophils, natural killer (NK) cells and T cells (12, 17, 18). For example, testosterone reduces inflammatory reactions such as toll-like receptor (TLR)4 expression on neutrophils, the biosynthesis of TNFα and iNOS-derived NO in macrophages, and NK cell activity (i.e., interferon (INF)γ secretion). On the other hand, androgens can promote anti-inflammatory processes such as expression of peroxisome proliferator-activated receptor (PPAR)α in T cells as well as IL-10 and transforming growth factor (TGF)β formation (11, 18).

Sex differences in PG formation were reported in 1972, where male human subjects were found to excrete larger amounts of the major urinary metabolite 7α-hydroxy-5, 11-diketotetranor-prostane-1, 16-dioic acid of PGE_1/2_ than female subjects (19). Later, in 1974, androgens were found to modulate PG formation (20, 21). In 2008, a striking suppressive potential of androgens on LT formation in human neutrophils was discovered that is causative for lower LT biosynthesis in male vs. female cells (22). Subsequent intensive research in humans and rodents has accumulated substantial knowledge on the impact of androgens on LM biology, and how this translates into modulation of inflammation, and how it affects anti-inflammatory therapy (23). Here we review the effects of androgens on pro-inflammatory LM biosynthesis and we discuss the role of LM in the interplay between androgen functions in innate immune cells with apparent consequences for the incidence and severity of inflammation.

## Lipid Mediators: Biosynthesis Pathways and Their Role in Inflammation

Innate immune cells including granulocytes, monocytes and macrophages possess high capacities to generate pro-inflammatory but also inflammation-resolving LM. Because of their potent bioactivities and broad impact on multiple functions of the body, LM are not stored in cells or tissues at large amounts but are rather *de novo*-synthesized on demand. The first step in the biosynthesis of LM is the liberation of the fatty acid substrate mainly from membrane phospholipids by phospholipase A_2_ (PLA_2_) enzymes (8, 24). Thus, modulation of PLA_2_ and concomitant fatty acid release as LM substrate may be subject of regulation by androgens and/or sex. However, only little is known on androgens affecting PLA_2_ (25, 26) and how this would translate into significant biological effects or sex differences connected to LM.

**Figure 1:**
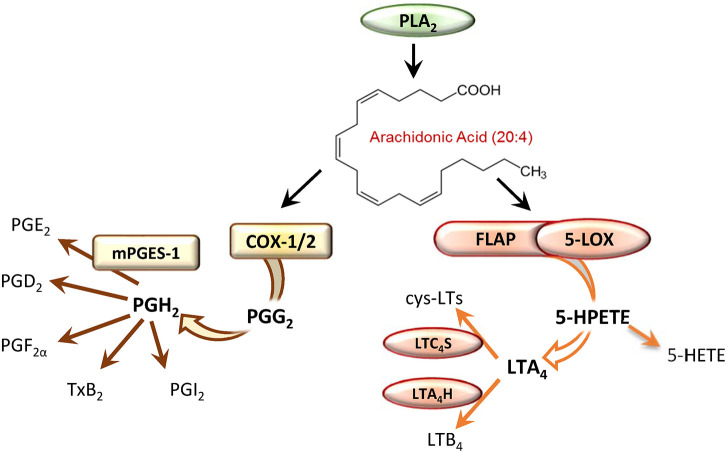
Enzymatic conversion of arachidonic acid by the cyclooxygenase (COX) or the 5-lipoxygenase (5-LOX) pathway leads to formation of prostaglandins (PG) or leukotrienes (LT), respectively.

PGs and thromboxanes (TX)s are generated by COX-1/2 in a two-step conversion of liberated AA which leads to the formation of the instable intermediates PGG_2_ and then PGH_2_, where the latter is transformed by tissue-specific prostanoid synthases into the different bioactive prostanoids PGD_2_, PGE_2_, PGF_2α_, PGI_2_, and TXA_2_ (Figure 1). Two isoforms of COX exist with COX-1 being constitutively expressed in many different cell types, and the inducible COX-2, which is abundantly expressed in pro-inflammatory innate immune cells including monocytes, neutrophils and M1-like macrophages after exposure to inflammatory stimuli (e.g., cytokines, bacterial components or hormones). PGE_2_ mediates inflammation-related processes such as vasodilatation and increased microvascular permeability, as well as fever, and pain (27). It is synthesized from PGH_2_ by cytosolic PGE synthase (cPGES) or microsomal PGE synthases (mPGES-1 and mPGES-2), with mPGES-1 being an inducible isoform with high relevance for producing PGE_2_ during inflammation (28).

LTs have significant impact on inflammatory and immune-related diseases like asthma and allergic rhinitis, autoimmune diseases, atherosclerosis, cardiovascular diseases and cancer (29). The ability to generate LTs is mainly restricted to innate immune cells such as neutrophils, eosinophils, monocytes, macrophages and mast cells that express the required biosynthetic enzymes. For the biosynthesis of LTs, AA is released from phospholipids by the cytosolic PLA_2_ and provided to 5-LOX by the nuclear membrane-bound 5-LOX-activating protein (FLAP) (30). 5-LOX then facilitates the incorporation of molecular oxygen at C5 of AA yielding the hydroperoxide 5(S)-HPETE that is converted in a second step by 5-LOX into the epoxide LTA_4_ (Figure 1). 5-LOX is localized in the nucleoplasm or the cytosol depending on the cell type, and after cell activation it translocates to the nuclear envelope in order to access liberated AA (31). 5-LOX is composed of a catalytic domain, with an iron in the active site, and a regulatory C2-like domain that binds Ca^2+^ and mediates the association with phosphatidylcholine (PC) and thus with PC-rich membranes (30). At the nuclear membrane 5-LOX assembles a complex with FLAP that binds AA and facilitates the accessibility of the substrate for 5-LOX, which is considered pivotal for LTA_4_ generation in intact cells (32, 33). Depending on the cell-type and the enzymes expressed, LTA_4_ is then converted to LTB_4_ by LTA_4_ hydrolase (LTA_4_H) or to LTC_4_ by LTC_4_ synthase (LTC_4_S) (30) (Figure 1). LTB_4_ is a potent chemoattractant and activator of various leukocytes whereas cys-LTs mediate smooth muscle contraction and vascular leakage (8).

In contrast to pro-inflammatory PGs and LTs, the SPM have anti-inflammatory properties as they actively terminate inflammation and promote the resolution of the inflammation process. The SPM superfamily encompasses LM with conserved structures mediating their bioactions via distinct GPCR with defined structure-activity relationships (2, 7). SPMs exhibit beneficial functions in microbial defense, pain, organ protection, and tissue regeneration, wound healing, cancer, reproduction, and neurobiology of cognition (9). The generation of LX from AA requires the concomitant actions of 5-LOX and 12/15-LOXs which can be accomplished by single cells that express both LOXs or by transcellular metabolism between cells that express at least one of these enzymes (9, 34). Similarly, the formation of Rv from EPA or DHA depends on at least two enzymes: a CYP450 or COX-2 in the presence of aspirin first generate 18-HEPE from EPA, while in a second step 18-HEPE is converted by a LOX yielding E-series Rv, or in the case of D-series Rv transformation of DHA by 15-LOX to 17-HDHA and its subsequent conversion by 5-LOX and other enzymes (34–36). On the other hand, for the biosynthesis of PD and MaR from DHA, 15-LOX and/or 12-LOX appear sufficient (37). Finally, stereoisomers of LX, Rvs and PD, so-called aspirin-triggered (AT)-LXs, AT-Rvs, and AT-PDs account to the SPM superfamily and are produced by LOXs alone or in conjunction with aspirin-induced acetylated COX-2 (9, 34). To the best of our knowledge, effects of androgens on the biosynthesis of SPM have not been reported yet. Nevertheless, sex differences in SPM levels in humans and mice were recently documented (38–41). Along these lines, the impact of androgens on 12- and 15-LOXs in innate immune cells is essentially unexplored; one study reported that androgen failed to alter 15-LOX-2 gene expression in normal human prostate epithelial cells (42).

## Sex Differences in Inflammation and in Autoimmune Diseases

Numerous studies have shown that sex is a significant variable in the activity of the innate and the adaptive immune system and that there are significant sex differences in the immune response (11, 43). This affects the defense against infections, autoimmune diseases and tumor diseases as well as vaccinations. Adult women generally have a more active innate and adaptive immune system, explaining why 80% of patients with autoimmune diseases are females, and why women with acute HIV infection have 40% less viral RNA in the blood than men. Moreover, antibody formation in response to influenza vaccines is consistently at least twice as high in women than in men. Age plays a decisive role in this, according to which the immune response and inflammatory activity predominate until puberty in boys, but then dominate in adult women (12). Since sex differences are often caused by divergent levels of androgens, many studies that revealed modulation of the innate immunity by male sex hormones originate from studies primarily aiming to compare biological responses in male and female subjects.

Studies on the mechanisms underlying sex-biased immune responses show quite complex differences in various immune cells between the sexes, both with regard to the innate and the adaptive immunity. For innate immunity, the phagocytosis rate of neutrophils and macrophages, the macrophage activity, the type 1 interferon activity of dendritic cells (DC), and the efficiency of antigen-presenting cells (APC) are all more pronounced in female vs. male cells (44–46). In contrast, the numbers of natural killer cells and the expression of the TLR4 of macrophages and neutrophils dominate in men (11). However, TLR7 is more strongly expressed in women (47). In terms of acquired immunity, women have more B lymphocytes and, accordingly, more pronounced antibody production, higher numbers of activated T lymphocytes with a higher proliferation rate, more CD4+ cells, and more pronounced T cell cytotoxicity. Men, on the other hand, have higher numbers of regulatory T lymphocytes and CD8+ cells (11).

Due to the higher activity of the immune system in women, inflammatory reactions occur more frequently and more severely in females than males. Distinct sex differences can be found in the incidence of autoimmune diseases such as multiple sclerosis (MS), systemic lupus erythematosus (SLE), thyroid disease, scleroderma or rheumatoid arthritis, but also Alzheimer's disease, which mostly dominate in women (48). In the US, women represent ~80% of all cases of autoimmune disorders. Also in animal models of autoimmune disease such as experimental autoimmune encephalomyelitis (EAE), females show more pronounced severity (49) with higher activation status of T_H_1 cells and elevated INFγ (50). Androgens are protective in the susceptibility to EAE of SJL mice, as castration caused increased severity of the disease and supplementation with exogenous testosterone to both gonadally intact SJL and C57BL/6 mice resulted in protecting effects. Of note, ovariectomy did not alter the disease outcomes (51). Interestingly, administration of sex hormones to patients with MS revealed beneficial effects: in men, topical application of testosterone significantly reduced IL-2 but elevated TGFβ production in PBMC, and shifted the lymphocyte composition by decreasing CD4+ T cells and increasing NK cells (52).

## Sex Bias in Prostaglandin and Leukotriene Biosynthesis

More than 50 years ago, modulation of eicosanoid biosynthesis by sex was reported (53) and effects of testosterone on PGF_2α_ formation were observed already in 1974 (20, 21). Until today, multiple studies employing different animal models uncovered sex differences and effects of sex hormones on the biosynthetic pathways of PGs and LTs in various cells and organs/tissues (Tables 1, 2), see also (66) for review. LM levels *in vivo* can be affected at the level of their biosynthesis as well as of their metabolism/elimination. Most studies addressed the regulation of the biosynthesis of LM, focusing on the expression of LOXs, COXs or prostanoid synthases as biosynthetic enzymes, or on the cellular activation of these enzymes (66). Notably, also the receptors that induce LM formation (e.g., TLR4) can be strongly modulated by sex and sex hormones (11).

**Table 1:** Sex differences and modulatory effects of androgens on leukotriene formation in various models and cell/tissue sources.

**Species**	**Model/Source**	**Stimulus**	**Effect of androgens**	**Male vs. Female**	**Reference**
Human	Whole blood	A23187 fMLP	⇓	M < F	(22, 23)
	Neutrophils	A23187	⇓	M < F	(22, 23)
	Monocytes	A23187	⇓	M < F	(54)
	Emotional tears	–	n.d.	M > F	(38)
Mouse	Peritonitis	zymosan	⇓	M < F	(23, 55)
	Peritoneal macrophages	A23187	n.d.	M < F	(55)
	Lungs	OVA	n.d.	M < F	(56)
Rat	Pleurisy	carrageenan	n.d.	M < F	(23)

**Table 2:** Sex differences and modulatory effects of androgens on prostaglandin formation in various models and cell/tissue sources.

**Species**	**Model/Source**	**Stimulus**	**Effect of androgens**	**Male vs. Female**	**References**
Human	Emotional tears	–	n.d.	M > F	(38)
	Neutrophils	A23187	n.d.	M > F	(57)
	Monocytes	LPS	⇓	n.d.	(58)
	Primary human coronary artery smooth muscle cells (HCASMC)	– LPS IL-1β	⇑ ⇓ ⇓	n.d.	(59)
	HUVECs	LPS TNFα	⇓ ⇓	n.d.	(60)
Mouse	Peritonitis	zymosan	n.d.	M > F	(57)
	Testis, epididymis, vas deferens, and seminal vesicles	–	⇓	n.d.	(61)
Rat	Pleurisy	carrageenan	n.d.	M > F	(57)
	Arthritis	collagen	⇓	M < F	(62)
	Vas deferens, epididymis and the seminal vesicles	–	⇑	n.d.	(20)
	Prostatic and vesicular glands	–	⇓	n.d.	(21)
	Cerebral blood vessels	–	⇑	n.d.	(63, 64)
	Bladder epithelium	–	⇓	n.d.	(65)

The existence of a sex dimorphism in LT biology is already suggested by the fact that many diseases related to LT including asthma, rheumatoid arthritis, allergic rhinitis, or SLE are sex-biased with higher occurrence in women (66). Similar as for PG formation, modulation of LT production may occur on the expression level of LT-biosynthetic enzymes and via the availability of AA as substrate but also additional regulatory aspects such as modulation of phosphorylation and subcellular redistribution of the biosynthetic enzymes. Note that despite comprehensive and intensive research with the aim to reveal roles of LTs in sex-afflicted autoimmune diseases such as SLE, potential sex differences in LT biosynthesis have long been neglected in biomedical research.

In fact, there is accumulating evidence suggesting that female sex is afflicted with higher LT biosynthesis and androgens were shown to lower LT levels *in vitro* and *in vivo* (Table 1) (22, 23, 54–56). For example, blood or isolated monocytes and neutrophils from healthy adult women exhibited higher capacities to produce LTs upon stimulation *ex vivo* vs. men (22, 23, 54). Also, in urine samples from healthy white volunteers, higher concentrations of 5-LOX products were found in samples from elderly women than elderly men (67). Such sex-bias was evident also in mice and rats *in vivo*, where female animals in zymosan-induced peritonitis, carrageenan-elicited pleurisy or experimental models of inflammation upon allergen challenge produced higher levels of LTs (23, 55, 56).

Studies with knock-out (KO) animals where 5-LOX or LT receptors were deleted revealed obvious roles of LTs for sex differences in the pathophysiology of diseases. For example, in SLE-related mouse models the female disadvantage vs. male animals was abolished after 5-LOX KO (68). Deletion of the BLT1 receptor protected only female but not male mice in platelet-activating factor (PAF)-induced shock (69) and knockout of the Cys-LT1 reduced tumor burden in the small intestine of female Apc (Min/+) mice but not in male counterparts (70). Conclusively, the superior benefit of female animals after disruption of the LT pathway implies the existence of sex differences in LT biosynthesis with more pronounced pathophysiological roles of LTs in females.

In line with the genetic interference with the 5-LOX pathway, also results from pharmacological studies intervening with LTs imply superior significance of LT biology in women, suggesting that females may benefit from anti-LT therapy to a greater extent than males. In a prospective cohort study with 11 asthmatic women and 10 men, montelukast [a potent and selective leukotriene receptor antagonist (71)] improved asthma symptoms and lung function better in women compared with men (72). Similarly, in asthmatic girls reaching puberty, montelukast clearly reduced asthma incidence vs. placebo, but not so in boys of the same age, even though montelukast was highly active in boys before puberty (73). Along these lines, superior therapeutic efficacy of different types of clinically relevant LT modifiers like zileuton, MK886 and montelukast were observed in female ovalbumin-sensitized BALB/c mice (56). Recent data showed that FLAP inhibitors like MK886 have impaired efficiency in males which is caused by androgens. Thus, lower doses of MK886 were needed to reduce LTB_4_ levels in inflammatory exudates of female vs. male mice and rats. Following PAF-induced shock, MK886 increased survival exclusively in female mice, which was abolished by androgen administration. FLAP inhibitors had higher potencies in human blood from females and supplementation of female blood with androgens abolished the observed sex differences (23). Together, the androgen-mediated suppression of LT biosynthesis is not only of pathophysiological relevance for LT-related diseases, but also determines the efficacy of anti-LTs, in particular the potency of FLAP inhibitors which is strikingly impaired by androgens.

Sex differences in PG formation are frequently related to sex-biased expression of the key enzymes involved. Thus, sex-related regulation of COX-2 and mPGES-1 in different organs with consequences for the development and incidence of PG-related diseases was reported (66). In general, prostanoids and their biosynthetic enzymes often dominate in males vs. females, but this can depend on the type of cell, tissue and organ, on the inflammatory status, as well as on the sex hormone level related to the age of the subjects studied. Most studies addressed kidney and brain, where in particular the COX-2/mPGES-1-derived PGE_2_ plays major physiological and pathophysiological roles. Three independent studies in rodents addressed sex differences in PG biology of the kidney indicating that female animals reached higher PG (mainly PGE_2_) concentrations vs. male counterparts. This bias was either due to superior COX-2 and mPGES-1 levels in females (74, 75) or due to higher expression of the PG-metabolizing enzyme 15-hydroxy-PG dehydrogenase (15-PGDH) and the PG-specific transporter OAT-PG in males (76). In contrast, in the brain, a preponderance of PG were evident in males (e.g., higher COX-2 expression), and suppressive effects by female sex hormones were reported (77–80). Most strikingly, under inflammatory conditions, for example in mouse zymosan-induced peritonitis and rat carrageenan-induced pleurisy, PG levels dominated in males vs. females, seemingly due to higher PG production in infiltrated male neutrophils that produced more PGE_2_ accompanied by elevated COX-2 expression (57). Note that sex differences in prostanoid biology may be caused also by a dimorphism in the expression and function of prostanoid receptors (66). Finally, sex differences in prostanoid biosynthesis and modulation by sex hormones were also observed on the cellular level where cells (e.g., neutrophils, platelets) from male donors generated more prostanoids as compared to female counterparts (38, 57, 81, 82).

## Impact of Androgens on Lipid Mediator Formation in Innate Immune Cells

### Androgens as Modulators of Innate Immune Cells

Among innate immune cells, neutrophils and monocytes/macrophages are the major source of LM, particularly in the blood. As reported below (section Androgens and Leukotriene Biosynthesis and Androgens and Biosynthesis of Prostaglandins and Other Eicosanoids), the influence of sex hormones and especially of androgens cause significant modifications in the production of LM with consequences for inflammation and possibly also the related pharmacology. There are several findings that indicate a considerable impact of androgens on the count and the functions of these LM-producing innate immune cells with consequences for the immune response. Neutrophils are generated in the bone marrow from myeloid progenitors and play a major role in the first response during inflammation as they represent the early cells recruited to the inflamed tissue to destroy external pathogens (83). It has been shown that the lack of androgen receptor (AR) causes neutropenia and increases the susceptibility to microbial infection (84). Also in a model of ozone-induced airway hyperresponsiveness, androgens caused an increased inflammatory response in terms of neutrophil recruitment as well as cytokine production in an AR-dependent manner where castration of mice diminished the effect (85). Accordingly, in a bacterial model of prostate inflammation, neutrophil accumulation in several organs and in the inflamed region was promoted by testosterone treatment (86). Note that different and contradictory effects of testosterone on neutrophil functions were reported: for example, after trauma-hemorrhagic shock, neutrophils from male mice were more activated then the female counterparts (87), while after bacteria-induced prostate inflammation, neutrophils from male animals produced less pro-inflammatory cytokines (85).

Monocytes and macrophages both express the AR and the effect of testosterone on the functions of those cells is widely discussed (18, 88). Monocytes account for up to ~10% of the circulating leukocytes in humans and ~4% in mice and they are evoked during inflammation in large amounts in order to amplify the inflammatory reaction (89). Macrophages that possess high plasticity, can act as pro-inflammatory (M1) or pro-resolving phenotypes (M2), the latter promoting the return to homeostasis (90). Intriguingly, testosterone reduces the expression of TLR4 in murine macrophages (91), but male macrophages have seemingly a superior response to TLR4 ligands (92). Testosterone may also impact macrophage polarization: for example, upon Coxsackievirus B infection, male mice suffered more from myocarditis than females which correlated with a predominance of M2 in female myocardium as compared to increased numbers of M1 in males (93).

### Androgens and Leukotriene Biosynthesis

The superior levels of LTs formed in women and female animals or in cells derived from female subjects vs. male counterparts can be unequivocally related to the suppressive impact of androgens. Table 1 summarizes the reported effects of androgens in various models in relation to the observed sex differences in these studies. The first report demonstrating modulation of LT biosynthesis by androgens *in vitro* was published in 2008 by Pergola et al. (22), almost 30 years after the discovery of LTs by Samuelsson et al. in 1979 (94). Suppression of 5-LOX product formation by androgens like testosterone or 5α-DHT was observed in agonist-stimulated human blood, isolated human neutrophils (22, 23) and monocytes (54) from females. Also in human corneal, conjunctival, and meibomian gland epithelial cells 5α-DHT reduced the potentiation of LPS-induced secretion of LTB_4_ via LPS-binding protein (95).

The existence of such androgen effects *in vivo* was confirmed in a murine zymosan-induced peritonitis model (55). Thus, LT levels in peritoneal exudates of orchidectomized mice were higher than in sham male mice, and peritoneal macrophages from orchidectomized animals produced more LTs *ex vivo* than sham-treated counterparts (55). Along these lines, short–term application of 5α-DHT reduced LTB_4_ levels during zymosan-induced peritonitis only in female but not in male animals (23). Androgen-mediated sex differences were recently shown also in a mouse model of MS (16), where LT signaling contributes to pathology (96). Moreover, *ex vivo* analysis of blood from healthy women with variant androgen levels revealed a significant invers correlation between androgen levels and the capacity to produce LTs upon stimulation of the blood (22).

In leukocytes, suppression of LT formation by androgens required very short pre-incubation periods of only one to few minutes, excluding the requirement of protein *de novo* synthesis, supported by the fact that androgen treatment did not alter the amounts of any of the LT biosynthetic enzymes on the protein level. Also, in neutrophils and monocytes derived from male and female subjects these LT biosynthetic enzymes were equally expressed (22, 54). Moreover, the magnitude of (concentration-dependent) maximal inhibition of LTs was <50% of the total 5-LOX activity, suggesting that androgens do not directly act on 5-LOX like typical enzyme inhibitors. In fact, androgens were not active in male blood or leukocytes and they suppressed 5-LOX product formation in female cells or blood down to the levels obtained in male counterparts. Interestingly, supplementation of these neutrophils or monocytes with exogenous AA as substrate reverted androgen-induced inhibition of 5-LOX, indicating that androgens may inhibit 5-LOX product formation at the level of substrate supply (20). However, 5α-DHT failed to inhibit AA release in these leukocytes, suggesting another point of attack for androgens; seemingly the accessibility of AA to 5-LOX mediated by FLAP is affected by the sex hormone.

For LT formation, soluble resting 5-LOX in the cytosol or nucleosol must translocate to the nuclear envelope upon cell stimulation for associating with FLAP that facilitates access of 5-LOX to endogenous AA as substrate (32, 33). While in female neutrophils, this pattern of 5-LOX subcellular redistribution was evident along with high LT levels, in male neutrophils a substantial part of 5-LOX was located to the nuclear envelope in resting cells, and only a small portion of 5-LOX redistributed upon stimulation, accompanied by low 5-LOX product biosynthesis (22). Treatment of female cells with 5α-DHT caused the same pattern of 5-LOX redistribution like in male neutrophils and suppressed LT biosynthesis in female cells down to the levels obtained by male neutrophils. Note that also cPLA_2_ is a cytosolic enzyme that co-translocates with 5-LOX to the nuclear envelope where AA as substrate for 5-LOX is liberated from PC (24). However, neither sex differences nor effects of androgens were observed for cPLA_2_ translocation in neutrophils (22), suggesting that the sex bias in LT formation is independent from cPLA_2_-mediated substrate supply. On the other hand, sex differences in *n*-3 PUFA content were reported with associated differences in circulating concentrations of *n*-3 PUFA, that is, higher plasma DHA concentrations in females and negatively associated with circulating concentrations of testosterone (83).

More detailed mechanistic analysis on the subcellular localization of 5-LOX uncovered that androgens impede the agonist-induced, tight assembly of the LT-biosynthetic 5-LOX/FLAP complex at the nuclear membrane of human and murine leukocytes (23). By means of a proximity ligation assay that enables the visualization of *in-situ* 5-LOX/FLAP interaction in intact cells (32) it was found that despite perinuclear localization of 5-LOX in male neutrophils, monocytes or peritoneal macrophages, 5-LOX and FLAP hardly associated as compared to female cells. Addition of 5α-DHT to female cells prevented the agonist-induced 5-LOX/FLAP complex assembly as it was observed in male cells (23). In conclusion, androgens may cause perinuclear localization of 5-LOX but distant from nuclear membrane-bound FLAP which impedes 5-LOX product formation.

It was shown that the variant testosterone levels in males and females cause a differential activation status of extracellular signal-regulated protein kinase (ERK)-1/2 in human neutrophils which confers sex differences in LT formation by regulating 5-LOX subcellular localization (22). Because ERKs are central signaling kinases that regulate multiple neutrophil functions, these actions of androgens may have striking effects on neutrophil biology besides regulating LT formation. 5-LO translocation is governed by ERK-1/2 that phosphorylate 5-LOX (97) and it was shown that ERK-1/2 mediate the male pattern of 5-LOX subcellular distribution caused by androgens. Thus, male (resting) neutrophils exhibited a higher ERK-1/2 activation status vs. cells from females, and pharmacological suppression of ERK-1/2 activity in male cells yielded the female 5-LOX subcellular localization pattern. Of interest, exposure of female neutrophils to male plasma or to 5α-DHT enhanced ERK-1/2 activation, accompanied by the male pattern of 5-LOX subcellular distribution, which again was reversed by ERK-1/2 inhibition (22). ERK activation in neutrophils by 5α-DHT was rather rapid, occurring with 0.5 min, at fairly low concentrations, starting at ~10 pM. Rapid activation of ERK-1/2 by androgens through a non-genomic mechanism was shown before for non-immune PMC42 breast cancer cells (98). Notably, the androgen effects on ERK-1/2 and 5-LOX were reversible and disappeared after about 1 h at room temperature but were preserved for longer times when neutrophils were kept at 4°C (22). This finding implies that isolation of neutrophil and probably also of other blood cells at low temperature, i.e., 4°C, may better maintain the biology of the cells that they once possessed in their original environment.

As mentioned above, in addition to neutrophils, higher 5-LOX product formation (about 1.8-fold) was also found for isolated human monocytes derived from peripheral blood of females vs. monocytes from males (54). Again, resuspension of female monocytes in plasma from males lowered 5-LOX product formation, and preincubation with 5α-DHT for 30 min repressed LT synthesis in female cells down to the levels observed in males, while estradiol and progesterone were inactive or gave only slight inhibition. 5α-DHT caused rapid ERK-1/2 phosphorylation connected to inhibition of phospholipase D (PLD) and reduced diacylglycerol (DAG) formation. It was shown that DAG are of importance for 5-LOX product formation (99) by acting at a phospholipid binding site of 5-LOX located within the C2-like domain (100). In fact, in male monocytes the ERK-1/2 activation status was increased while PLD activity and DAG formation was 1.4–1.8-fold lower vs. female monocytes. Supplementation of monocytes with DAG elevated 5-LOX product formation in male but not in female cells. These results indicate that androgen-induced ERK-1/2 activation represses PLD activity in monocytes resulting in impaired 5-LOX product formation seemingly due to lack of activating DAGs (54).

In addition to androgens, also progesterone was found to down-regulate LT formation in human monocytes in a rapid manner, however, not in neutrophils (101). Progesterone was more effective in monocytes derived from females and suppression of 5-LOX product synthesis was proposed to be mediated by protein kinase A, whereas ERK-1/2 and PLD were seemingly not involved (101), indicating that the characteristics and mechanisms behind the LT-suppressive effects of progesterone and androgens clearly differ. Moreover, estradiol was shown to induce LTC_4_ biosynthesis in RBL-2H3 cells and potentiated LTC_4_ formation in response to IgE seemingly by promoting intracellular Ca^2+^ mobilization (102). Finally, pregnancy, a situation with marked alterations in sex hormone plasma levels, was shown to be associated with increased LT biosynthesis in blood due to higher numbers of leukocyte and due to stimulatory effects of plasma components (103). It is interesting in this respect that the characteristics of diseases related to LT (e.g., asthma, allergic rhinitis) change during pregnancy (104).

### Androgens and Biosynthesis of Prostaglandins and Other Eicosanoids

The impact of sex hormones on PG biosynthesis has been considered in various types of preclinical experimental animal models and in clinical studies, where sex differences in prostanoid formation were evident. In contrast to modulation of the 5-LOX pathway, the underlying mechanisms of the effects of androgens on PG formation are less clear and will be addressed here only briefly. Many studies reported on effects of androgens on PG formation in rodents with divergent and even opposing outcomes that seemingly depend on the experimental settings and conditions (Table 2). In fact, the first studies analyzing PG formation in the reproductive system of male rats in relation to androgens were conflicting: Bartke and Koerner documented reduced PGF_2_α levels in the vas deferens, epididymis and the seminal vesicles of male rats upon castration which were reversed by administration of testosterone (20), while Sutherland et al. (21) found elevated PGF_2_α in prostatic and vesicular glands upon orchidectomy of male rats which was reversed by testosterone application. It seems that androgens differentially influence PG biosynthetic pathways under physiological and pathophysiological conditions in various tissues and act at different levels. As a rule of thumb, androgens appear to elevate PG formation by augmenting COX-2 expression under healthy states, but they suppress COX-2 induction and PG biosynthesis under inflammatory conditions (Table 2). Thus, in the absence of an inflammatory stimulus, supplementation of testosterone in orchidectomized male rats increased COX-2 protein expression in cerebral blood vessels along with elevated PGE_2_ levels (63, 64), in rat bladder epithelium (65), and PGE_2_ and COX-2 were elevated in primary human coronary artery smooth muscle cells (HCASMC) by 5α-DHT (59). Conversely, in the presence of LPS or IL-1β, the increases in COX-2 protein in HCASMC (59) or in human monocytes (58) were attenuated by testosterone, which was observed also in LPS- or TNFα-stimulated HUVECs where the elevated mRNA levels of COX-2 were repressed when 5α-DHT was present (60). Also, under collagen-induced arthritis in castrated female rats, testosterone application possessed significant anti-inflammatory effects accompanied by reduced PGE_2_ levels (62). While most of the PG-modulatory effects of androgens were connected to alterations in COX-2 levels, also the expression of mPGES-1 is seemingly affected by androgens. Thus, urinary PGE_2_ levels and mPGES-1 expression in the renal inner medulla of spontaneously hypertensive rats was lower in males but after orchidectomy, PGE_2_ metabolite excretion and mPGES-1 expression were elevated (75). Androgens may also affect the metabolism and transport of PG. For example, the PG-catabolic enzyme 15-PGDH and the PG-specific transporter OAT-PG are more abundant in the renal cortex of male rats with consequently lower renocortical PGE_2_. Orchidectomization of male rats led to decreased OAT-PG expression and elevate renocortical PGE_2_, and these changes were restored upon supplementation of testosterone (76).

Finally, androgens may impact the ω-hydroxylated AA metabolite 20-hydroxyeicosatetraenoic acid (20-HETE), generated by CYP enzymes, that mediates hypertension via multiple pathways. Several studies indicate that 20-HETE may play a role in androgen-induced vascular dysfunction and hypertension (105). Thus, treatment of normotensive mice with 5α-DHT induced both Cyp4a12 expression and 20-HETE levels in preglomerular microvessels, where 20-HETE can mediate both androgen-induced and androgen-independent hypertension (106). In an S6 mice strain, deficient in soluble guanylate cyclase-α1, hypertension was traced back to elevated Cyp4a12a expression and increased 20-HETE levels (107). Moreover, 20-HETE produced by CYP4a2 may contribute to elevated blood pressure in hyperandrogenic female rats (108).

## Concluding Remarks

A variety of inflammatory diseases related to increased LT and PG are sex-biased with often higher incidence and severity in females, e.g., rheumatoid arthritis and asthma. Androgen-mediated sex differences in inflammation and in the activity of related innate immune cells are now well-documented. Androgens have a significant impact on neutrophils, monocytes and macrophages which are the major sources of inflammation-related LM that are biosynthesized on demand. Obvious sex differences in the capacity to generate LM led to the discovery of regulatory roles of androgens. For LT biosynthesis, the situation in males and females is rather unambiguous with superior LT levels in females, and there are clear indications for throughout suppressive effects of androgens on LT formation in neutrophils, monocytes and macrophages from human and/or mice (or rats). Androgens reduce LT biosynthesis in various inflammation models related to LT *in vivo* which can translate into impaired efficiency of anti-LTs. Intracellular signaling routes involving ERK-1/2 and/or PLD were uncovered as effectors of androgens in this respect which eventually prevents the assembly of the LT-biosynthetic 5-LOX/FLAP complex. Future studies will be needed to address by which receptor and signaling pathway androgens activate ERK-1/2 and how ERK-1/2 as well as PLD and DAGs are connected to aberrant 5-LOX subcellular localization preventing access to FLAP. Moreover, evaluation of the effects of androgens and other sex hormones on SPM formation is warranted in future research along with the regulation of related biosynthetic enzymes such as 12- and 15-LOXs. Sex specific differences in PG biosynthesis and the impact of androgens may depend on the organ/tissue but also on the presence or absence of inflammatory status: under healthy conditions, androgens may increase PG formation along with elevated COX-2 induction, while in presence of an inflammatory stimulus androgens may repress PG biosynthesis. Together, it appears that androgens significantly impact pro-inflammatory LM formation in innate immune cells with direct consequences for pathophysiology but also for the pharmacotherapy of inflammation.

## Author Contributions

SP and OW wrote the manuscript and prepared the figures and tables. All authors contributed to the article and approved the submitted version.

## Conflict of Interest

The authors declare that the research was conducted in the absence of any commercial or financial relationships that could be construed as a potential conflict of interest.
